# Author Correction: Versatile spaceborne photonics with chalcogenide phase-change materials

**DOI:** 10.1038/s41526-024-00384-6

**Published:** 2024-03-26

**Authors:** Hyun Jung Kim, Matthew Julian, Calum Williams, David Bombara, Juejun Hu, Tian Gu, Kiumars Aryana, Godfrey Sauti, William Humphreys

**Affiliations:** 1 NASA Langley Research Center, Hampton, VA USA; 2 Booz Allen Hamilton, Arlington, VA USA; 3 Department of Physics, University of Cambridge, Cambridge, CB3 0HE UK; 4 John A. Paulson School of Engineering and Applied Sciences, Harvard University, Cambridge, MA USA; 5 Department of Materials Science & Engineering, Massachusetts Institute of Technology, Cambridge, MA USA; 6 Materials Research Laboratory, Massachusetts Institute of Technology, Cambridge, MA USA

**Keywords:** Materials for optics, Aerospace engineering, Optical materials and structures

Correction to: *npj Microgravity* 10.1038/s41526-024-00358-8, published online 20 February 2024

In this article, the main text in the ‘Author contributions’ section has been replaced with the revised statement. The original article has been corrected.

